# A Proposed Framework for Implementing a Rights-Based Approach to Gerontological Education and Training

**DOI:** 10.1093/geroni/igab046.1314

**Published:** 2021-12-17

**Authors:** Dana Bradley, Laura Allen

**Affiliations:** 1 UMBC Erickson School of Aging Studies, Baltimore, Maryland, United States; 2 Bar-Ilan University, Ramat Gat, Tel Aviv, Israel

## Abstract

This paper uses the nine general principles that underpin human rights (Non-discrimination, Respect, Dignity, Autonomy, Equality, Self-fulfillment and Personal development, Full and effective participation, Intergenerational solidarity, and Recognition of intrinsic value and worth as a human being) to frame a right’s based approach. This framework looks beyond the older person and the issues they are facing to the structure and culture of the society itself and the ways in which it is contributing to challenges. Using this lens, we will discuss how to develop definitions and standards of right’s- based education that are culturally and contextually appropriate, define right’s based competencies and recognize, that despite the universal rights of older persons, the implementation may need to be adjusted for unique sociocultural environments. . Lastly we will outline a strategy to identify and train multidisciplinary teaching and research teams using this proposed framework.

